# Photoprotective Potential of a Yeast/Rice Fermentation Filtrate and Sialic Acid in Mitigating UVA-Induced Oxidative Stress and Mitochondrial Dysfunction in Skin Fibroblasts

**DOI:** 10.3390/molecules31081262

**Published:** 2026-04-11

**Authors:** Fan Yang, Mingxuan Li, Yao Zuo, Miao Guo, Zhi Liu, Hua Wang

**Affiliations:** 1 Department of Biotechnology, College of Life Science and Technology, Huazhong University of Science and Technology, Wuhan 430074, China; yangfanfenix@outlook.com; 2 Research & Development Center, Mageline Biology Tech Co., Ltd., Wuhan 430206, China; guomiao@mageline.cn; 3 Research and Development Center, Wuhan CASOV Green Biotech Co., Ltd., Wuhan 430071, China; mingxuan_li@cabio.cn (M.L.); freya.zuo@casov.net (Y.Z.)

**Keywords:** yeast/rice fermentation filtrate, sialic acid, dermal fibroblasts, UVA-induced oxidative stress, mitochondrial protection, photoprotection

## Abstract

Ultraviolet A (UVA) radiation induces oxidative stress and mitochondrial dysfunction in dermal fibroblasts, contributing to photodamage and skin aging. This study investigated the protective effects of Yeast/rice fermentation filtrate (RFF) and sialic acid (SA), both individually and in combination, against UVA-induced damage in human dermal fibroblasts. Cell viability, reactive oxygen species (ROS) levels, intracellular ATP and NAD^+^ contents, and mitochondrial membrane potential (ΔΨm) were evaluated following treatment. RFF, SA, and their combination significantly improved cell viability in UVA-damaged fibroblasts and reduced ROS generation. Notably, the combined treatment increased intracellular ATP levels by 201.2% (*p* < 0.05), with enhancements of 62.3% and 285.4% compared to RFF and SA alone, respectively. Additionally, the combined treatment significantly restored NAD^+^ levels and effectively preserved mitochondrial membrane potential. Transcriptomic analysis revealed modulation of pathways related to cellular energy metabolism, particularly AMPK, and upregulation of SIRT1, SIRT3, and SIRT5 expression. The RFF–SA combination confers robust UVA photoprotection by enhancing mitochondrial resilience, providing a foundation for the development of protective cosmetic formulations.

## 1. Introduction

Skin photoaging, characterized by wrinkles, loss of elasticity, and pigmentation changes, is primarily induced by ultraviolet A (UVA) radiation [1]. UVA penetrates the dermis and directly affects dermal fibroblasts, the key cells responsible for maintaining extracellular matrix integrity and collagen homeostasis [2,3,4]. The photodamage caused by UVA involves multiple interconnected mechanisms, including excessive reactive oxygen species (ROS) generation, mitochondrial dysfunction, and degradation of collagen and other matrix proteins, ultimately compromising skin structure and function [5,6]. Despite extensive research, the complexity of these molecular pathways continues to challenge the development of effective photoprotective strategies.

Oxidative stress and mitochondrial impairment play central roles in UVA-induced fibroblast damage [7,8,9]. Specifically, UVA exposure markedly elevates intracellular ROS, which induces oxidative damage to cellular components and disrupts mitochondrial integrity [10,11]. This disruption is characterized by loss of mitochondrial membrane potential (ΔΨm), decreased ATP synthesis, and alterations in mitochondrial morphology, including fragmentation and swelling, collectively impairing bioenergetic efficiency and cellular repair mechanisms [12,13,14]. The resulting energy deficit compromises collagen synthesis and extracellular matrix remodeling, while the release of cytochrome c and other pro-apoptotic factors promotes fibroblast senescence and apoptosis [15,16]. Therefore, strategies that restore mitochondrial function by stabilizing ΔΨm, enhancing ATP production, and preserving mitochondrial ultrastructure, together with reinforcing antioxidant defenses, are critical for mitigating photoaging [17,18,19]. Bioactive compounds capable of simultaneously alleviating oxidative stress and supporting mitochondrial bioenergetics hold considerable potential for enhancing dermal fibroblast resilience under UVA-induced stress.

Natural bioactive compounds have garnered increasing interest as promising photoprotective agents [20,21,22]. Yeast/Rice Fermentation Filtrate (RFF) contains a variety of low-molecular-weight bioactive components, including amino acids and peptides, which contribute to its moisturizing, barrier-strengthening, antioxidant, anti-inflammatory, and anti-aging effects [23,24]. Sialic acids (SAs), a family of acidic monosaccharides, similarly exhibit cytoprotective, antioxidant, and anti-inflammatory activities that support cellular integrity under stress [25,26]. The complementary biological activities of RFF and SA provide a rationale for their combination. Previous studies indicate that their combination more effectively inhibits inflammatory markers and enhances collagen accumulation through TGF-β/Smad-mediated transcriptional regulation and HuR-dependent mRNA stabilization [27,28]. However, while the individual photoprotective properties of RFF and SA are documented, their combined efficacy against UVA-induced damage in dermal fibroblasts remains poorly characterized. Investigating the interactions between these two bioactive agents could provide valuable insights for developing strategies to mitigate photoaging by simultaneously reducing oxidative stress, controlling inflammation, and supporting mitochondrial and collagen homeostasis at the cellular level.

In this study, human dermal fibroblasts were used because UVA radiation penetrates into the dermis and directly induces oxidative stress and mitochondrial dysfunction, which are central processes in skin photoaging. We systematically evaluated the photoprotective effects of RFF and SA, both individually and in combination, against UVA-induced damage in human dermal fibroblasts. Our investigation focused on their capacity to mitigate oxidative stress, restore mitochondrial function, and modulate cellular energy metabolism. Transcriptomic analysis was further performed to elucidate the underlying molecular mechanisms. The findings clarify the coordinated pathways through which RFF and SA enhance mitochondrial resilience and improve cellular bioenergetic homeostasis, providing a theoretical and experimental foundation for developing mitochondrion-targeted combined photoprotective formulations.

## 2. Materials and Methods

### 2.1. Chemicals and Reagents

Rice Fermentation Filtrate (RFF) was supplied by Mageline Biology Tech Co., Ltd. (Wuhan, China). Sialic acid (SA) was bought from Wuhan CASOV Green Biotech Co., Ltd. (Wuhan, China). Human dermal fibroblasts (HDFs; CRL-2845) were obtained from the Cell Bank of the Chinese Academy of Sciences (Shanghai, China). Dulbecco’s Modified Eagle Medium (DMEM, high glucose) and fetal bovine serum (FBS) for cell culture were supplied by Gibco. Trypsin was procured from HyClone. The following assay kits and reagents were used: Cell Counting Kit-8 (CCK-8, Beyotime, Shanghai, China) for measuring cell metabolic activity, 2′,7′-dichlorofluorescin diacetate (DCFH-DA) for reactive oxygen species detection (Beyotime, Shanghai, China), ATP Assay Kit (Beyotime, Shanghai, China), NAD^+^/NADH Assay Kit (Nanjing Jiancheng Bioengineering Institute, Nanjing, China), Mitochondrial Membrane Potential Assay Kit (JC-1, Beyotime, Shanghai, China), BCA Protein Assay Kit (Aspen, Beijing, China) for protein concentration determination, and DAPI staining solution (Solarbio, Beijing, China) for nuclear labeling.

### 2.2. Cell Culture and UVA Irradiation

Human dermal fibroblasts were cultured in high-glucose DMEM supplemented with 10% fetal bovine serum (FBS) and 1% penicillin-streptomycin at 37 °C in a humidified atmosphere containing 5% CO_2_. When cells reached approximately 80% confluence, they were detached using 0.25% trypsin, centrifuged, and resuspended. Cell density was determined using a hemocytometer (Thermo Fisher Scientific, Waltham, MA, USA). Based on the experimental design, cells were seeded at appropriate densities for each experimental group. For assays, cells were serum-starved for either 24 or 48 h, and exposed to a single UVA dose of 10 J/cm^2^ (Philips TL/10R lamps, 365 nm peak, measured with a Waldmann UV meter) [29]. Immediately after irradiation, the medium was replaced with fresh serum-free DMEM containing the test compounds. Five experimental groups were set up in triplicate: Control—no UVA, no additives; UVA—vehicle only; UVA + 0.025% *w*/*v* RFF; UVA + 0.5% *w*/*v* SA; UVA + 0.025% *w*/*v* RFF + 0.5% *w/v* SA. Cells were incubated for 24 h (for viability assays) or 48 h (for RNA isolation).

### 2.3. CCK-8 Assay

Human dermal fibroblasts were seeded into 96-well plates at a density of 1 × 10^4^ cells per well in 100 μL of culture medium and incubated overnight at 37 °C under 5% CO_2_ to allow for adherence. The following day, the medium was replaced with fresh medium containing the respective treatment agents, and cells were incubated for 24 h or 48 h. Except for the control group, all other groups were then exposed to UVA irradiation, followed by an additional 24 h incubation period. Subsequently, 10 μL of CCK-8 reagent (final concentration 10% *v*/*v*, Dojindo, Kumamoto, Japan) was added to each well, and plates were incubated for 2–4 h. Absorbance at 450 nm was measured using a microplate reader (CMax Plus, Molecular Devices, San Jose, CA, USA) to evaluate changes in cell viability. The CCK-8 assay protocol followed the manufacturer’s instructions [30].

### 2.4. Measurement of Intracellular Production of ROS

Cells were seeded into glass-bottom 24-well plates at a density of 5 × 10^4^ cells per well and incubated overnight at 37 °C under 5% CO_2_ to allow for adherence. The following day, the medium was replaced with fresh medium containing the respective treatment agents, and cells were incubated for 24 h or 48 h. Except for the control group, all other groups were exposed to UVA irradiation, followed by an additional 24 h incubation period. After treatment, the medium was removed, and cells were incubated with serum-free medium containing 10 μmol/L DCFH-DA at 37 °C for 30 min in the dark [31]. Following incubation, cells were washed three times with PBS to remove excess probe. Images were captured using an inverted fluorescence microscope, with at least three random fields recorded per group under consistent acquisition parameters. The mean fluorescence intensity per field was quantified using ImageJ software (version 1.53, National Institutes of Health, Bethesda, MD, USA) to reflect changes in intracellular ROS levels.

### 2.5. ATP Measurement

Cells were seeded into 96-well plates at a density of 1 × 10^4^ cells per well and incubated overnight at 37 °C under 5% CO_2_ to allow for adherence. The following day, the medium was replaced with fresh medium containing the respective treatment agents, and cells were incubated for 24 h or 48 h. Except for the control group, all other groups were exposed to UVA irradiation, followed by an additional 24 h incubation period. After treatment, the medium was discarded, and intracellular ATP content was determined according to the manufacturer’s instructions of the ATP Assay Kit (Beyotime, Shanghai, China) [32]. Briefly, after cell lysis, an equal volume of ATP detection reagent was added to each well, and the plates were incubated in the dark at room temperature for 10 min. Luminescence was then measured using a microplate luminometer (CMax Plus, Molecular Devices, San Jose, CA, USA). An ATP standard curve was included in each assay to ensure accuracy. In parallel, total protein concentration was measured using the BCA Protein Assay Kit according to the manufacturer’s protocol, with absorbance read at 562 nm using the CMax Plus microplate reader. ATP values were normalized to total protein content. All experiments were performed in triplicate.

### 2.6. NAD^+^ Measurement

Cells were seeded into 96-well plates at a density of 1 × 10^4^ cells per well and incubated overnight at 37 °C under 5% CO_2_ to allow for adherence. The next day, the medium was replaced with fresh medium containing the respective treatment agents, and cells were incubated for 24 h or 48 h. Except for the control group, all other groups were exposed to UVA irradiation, followed by an additional 24 h incubation period. After treatment, the medium was removed, and intracellular NAD^+^ levels were measured according to the manufacturer’s instructions of the NAD^+^/NADH Assay Kit (Beyotime, Shanghai, China) [32]. Briefly, cells were collected and lysed, and the supernatant was collected after centrifugation. Following the kit protocol, acid extraction was performed to separate NAD^+^. Chromogenic or luminescent reaction solution was then added, and the mixture was allowed to react at room temperature for the specified duration. Absorbance at 570 nm was measured using a CMax Plus microplate reader, and NAD^+^ content in each group was calculated accordingly.

### 2.7. Measurement of Mitochondrial Membrane Potential

Cells were seeded into glass-bottom 24-well plates at a density of 5 × 10^4^ cells per well and incubated overnight at 37 °C under 5% CO_2_ to allow for adherence. The next day, the medium was replaced with fresh medium containing the respective treatment agents, and cells were incubated for 24 h or 48 h. Except for the control group, all other groups were exposed to UVA irradiation, followed by an additional 24 h incubation period. After treatment, the medium was removed, and cells were incubated with 500 μL of JC-1 working solution (5 μg/mL, prepared according to the manufacturer’s instructions) at 37 °C for 20 min in the dark [33]. Following staining, cells were gently washed three times with PBS to remove unbound dye. Subsequently, cells were stained with PBS containing 1 μg/mL DAPI for 5 min at room temperature in the dark, and then washed again three times with PBS. Images were acquired using an inverted fluorescence microscope, with at least three random fields captured per group. The red and green fluorescence intensities were quantified using ImageJ software.

### 2.8. RNA Extraction and Transcriptome Sequencing

Total RNA was isolated using TRIzol reagent and treated with DNase I to remove genomic DNA. RNA integrity was assessed using an Agilent 2100 Bioanalyzer, and samples with an RNA Integrity Number (RIN) ≥ 7 were used for further analysis. RNA libraries were prepared using the NEBNext Ultra RNA Library Prep Kit (New England Biolabs, Ipswich, MA, USA) and sequenced on the Illumina NovaSeq 6000 platform (2 × 150 bp, ≥20 million clean reads per sample). Raw reads were processed and filtered using fastp, and then aligned to the GRCh38 reference genome using HISAT2. Gene-level read counts were generated using FeatureCounts (version 2.0.1). Differential expression analysis was performed using DESeq2 with a threshold of |log_2_FC| ≥ 1 and FDR < 0.05.

### 2.9. Reverse Transcription Quantitative Polymerase Chain Reaction (RT-qPCR)

cDNA was synthesised with PrimeScript RT Master Mix. qPCR was performed on a CFX96 system (Bio-Rad, Hercules, CA, USA) using SYBR Green with gene-specific primers (SIRT1–SIRT5, housekeeping GAPDH). Relative expression was calculated using the 2^−ΔΔCt^ method. Technical triplicates were averaged and biological replicates (n = 3) were analyzed using one-way ANOVA followed by Tukey’s test (*p* < 0.05 considered significant).

### 2.10. Statistical Analysis

Statistical analysis was performed using GraphPad Prism software (version 9.0, GraphPad Software, San Diego, CA, USA). Data are presented as mean ± standard deviation (SD). Differences between groups were initially assessed by independent-samples t-test and further evaluated by one-way analysis of variance (one-way ANOVA). A *p*-value < 0.05 was considered statistically significant, *p* < 0.01 was considered highly significant, and *p* < 0.001 was considered extremely significant.

## 3. Results

### 3.1. Protective Effect of the Combination on UVA-Induced Fibroblast Viability

**Figure 1:**
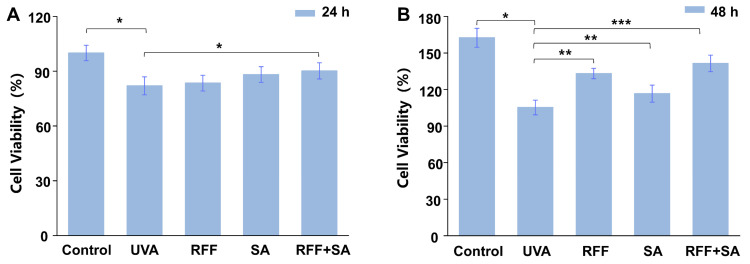
Effects of different pretreatments on HDF cell viability after 24 h (**A**) and 48 h (**B**). Cells were treated with control medium, UVA, Yeast/Rice Fermentation Filtrate (RFF), Sialic acid (SA), or the combination of RFF and SA (RFF+SA). Data are presented as the mean ± standard deviation (SD) from three independent experiments. * *p* < 0.05, ** *p* < 0.01, *** *p* < 0.001 compared with the control group.

To evaluate the overall cytoprotective effect of the combination under UVA-induced stress conditions, fibroblast viability was first assessed. As shown in Figure 1, UVA irradiation at 10 J/cm^2^ significantly reduced cell viability (*p* < 0.05). Following 24 h of pretreatment, neither 0.025% RFF nor 0.5% SA alone significantly restored cell viability; however, their combined treatment increased viability by 10.1% compared with the UVA group (*p* < 0.05). Upon extension of the pretreatment period to 48 h, RFF and SA administered individually enhanced cell viability by 26.5% and 10.8%, respectively (*p* < 0.05), whereas the combined treatment resulted in a more pronounced increase of 34.4% (*p* < 0.01). Collectively, these findings demonstrate that both RFF and SA confer photoprotective effects against UVA-induced cytotoxicity, while their combination exhibits superior efficacy in preserving fibroblast viability.

### 3.2. Attenuation of UVA-Induced ROS Generation by RFF and SA

**Figure 2:**
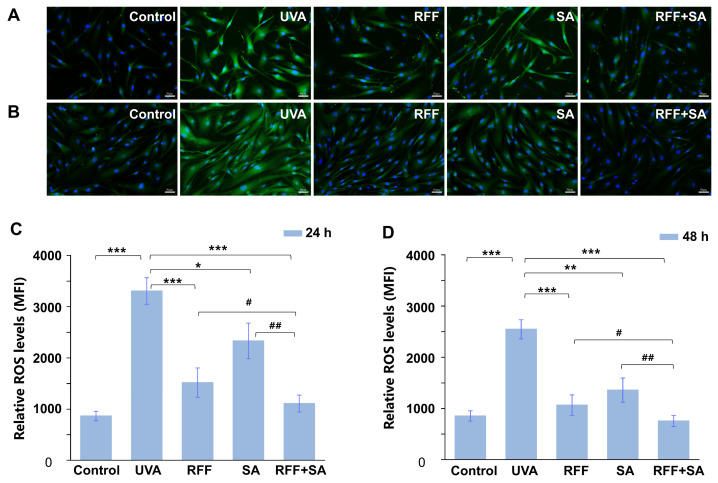
Attenuation of UVA-induced reactive oxygen species (ROS) generation by RFF and SA in HDF cells. Representative fluorescence images of intracellular ROS detected by DCFH-DA staining after pretreatment with RFF, SA, or their combination (RFF+SA) for 24 h (**A**) or 48 h (**B**), followed by UVA exposure. Green fluorescence indicates ROS levels, while blue fluorescence (DAPI staining) represents cell nuclei. Quantitative analysis of intracellular ROS levels after 24 h (**C**) and 48 h (**D**) pretreatment. Data are presented as mean ± SD (n = 3). * *p* < 0.05, ** *p* < 0.01, *** *p* < 0.001 versus the UVA group; ^#^ *p* < 0.05, ^##^ *p* < 0.01 versus the RFF or SA treatment groups.

As oxidative stress is a key contributor to UVA-induced cellular damage, intracellular ROS were quantified to evaluate the antioxidant capacity of the treatments. ROS production was assessed using the DCFH-DA fluorescent probe, which is hydrolyzed to nonfluorescent DCFH and subsequently oxidized to fluorescent DCF, with fluorescence intensity reflecting ROS accumulation. As shown in Figure 2A,B, UVA irradiation markedly increased green fluorescence intensity, indicating elevated ROS production and enhanced oxidative stress. In contrast, pretreatment with RFF, SA, or their combination for 24 h or 48 h prior to UVA exposure significantly reduced fluorescence intensity, suggesting effective suppression of ROS generation.

Quantitative analysis revealed that, after 24 h of pretreatment, intracellular ROS levels were reduced by 54.1%, 29.4%, and 66.4% in the RFF-, SA-, and combination-treated groups, respectively, compared with the UVA-exposed group (Figure 2C). Notably, the combination treatment resulted in significantly lower ROS levels than either RFF or SA alone (*p* < 0.05 and *p* < 0.01, respectively). Upon extending the pretreatment duration to 48 h, ROS levels were further decreased in all treated groups, with reductions of 58.1% for RFF, 46.6% for SA, and 70.3% for the combined treatment (Figure 2D). Again, the combination group exhibited the most pronounced reduction in ROS levels, differing significantly from both single-agent treatments.

### 3.3. Protective Effects Against UVA-Induced ATP Depletion

**Figure 3:**
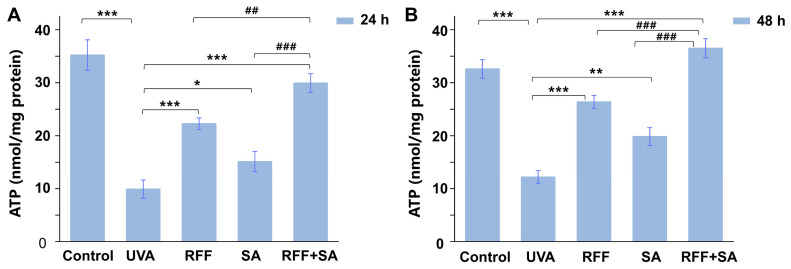
Effects of RFF, SA, and their combination on ATP production in UVA-exposed HDF cells. Intracellular ATP levels were measured after pretreatment with RFF, SA, or their combination (RFF+SA) for 24 h (**A**) or 48 h (**B**), followed by UVA irradiation. Data are expressed as mean ± SD (n = 3). * *p* < 0.05, ** *p* < 0.01, *** *p* < 0.001 versus UVA group; ^##^ *p* < 0.01, ^###^ *p* < 0.001 versus the RFF or SA treatment groups.

Given that oxidative stress-induced mitochondrial dysfunction often results in impaired energy production, ATP content was further examined. As a key indicator of cellular energy metabolism and physiological status [34,35], ATP levels were markedly reduced following UVA irradiation, indicating significant disruption of energy homeostasis (Figure 3). After 24 h of pretreatment, RFF and SA individually increased ATP levels by 124.0% and 52.2%, respectively, compared with the UVA group. Notably, combined pretreatment with RFF and SA resulted in a more substantial elevation of ATP levels (201.2%), corresponding to increases of 62.3% and 285.4% relative to RFF or SA alone, thereby demonstrating a combined enhancement of energy restoration. When the pretreatment duration was extended to 48 h, ATP levels in the combination group remained significantly elevated (199.8% versus the UVA group), exceeding those observed in both single-treatment groups and even surpassing ATP levels in the untreated control group.

### 3.4. Protective Effects Against UVA-Induced NAD^+^ Depletion

**Figure 4:**
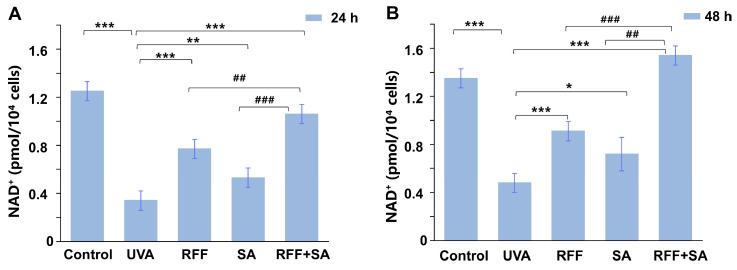
Effects of RFF, SA, and their combination on NAD^+^ levels in UVA-exposed HDF cells. Intracellular NAD^+^ content was measured after pretreatment with RFF, SA, or their combination (RFF+SA) for 24 h (**A**) or 48 h (**B**), followed by UVA irradiation. Data are expressed as mean ± SD (n = 3). * *p* < 0.05, ** *p* < 0.01, *** *p* < 0.001 versus UVA group; ^##^ *p* < 0.01, ^###^ *p* < 0.001 versus the RFF or SA treatment groups.

In line with the observed restoration of ATP levels, NAD^+^, a central cofactor in cellular energy metabolism and mitochondrial function [36,37], was also evaluated following UVA exposure. As shown in Figure 4, UVA irradiation markedly decreased NAD^+^ content (*p* < 0.001), indicating disruption of metabolic homeostasis. After 24 h of pretreatment, RFF and SA individually increased NAD^+^ levels by 128.5% and 56.9%, respectively, compared with the UVA-exposed group. Combined treatment with RFF and SA further elevated NAD^+^ levels to 214.2%, corresponding to increases of 66.7% and 276.4% relative to RFF or SA alone, demonstrating a pronounced combined effect. Following 48 h of pretreatment, the combination group exhibited a 220.0% increase in NAD^+^ levels versus the UVA group, which was 14.2% higher than the untreated control and exceeded the levels observed with either single-agent treatment.

### 3.5. Protective Effects on Mitochondrial Membrane Potential (*Δ*Ψm)

**Figure 5:**
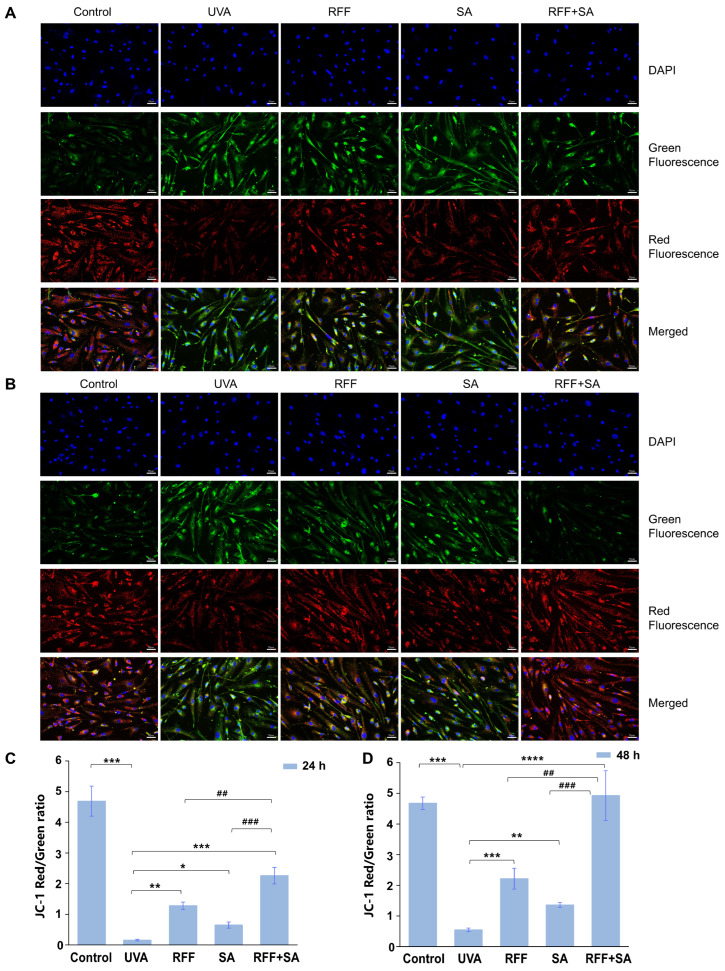
Effects of RFF, SA, and their combination on mitochondrial membrane potential (ΔΨm) in UVA-exposed HDF cells. Representative fluorescence images of JC-1 staining showing red fluorescence (J-aggregates, high ΔΨm) and green fluorescence (monomers, low ΔΨm) at (**A**) 24 h and (**B**) 48 h. Cells were pretreated with RFF, SA, or their combination (RFF+SA) for 24 h or 48 h prior to UVA irradiation. Blue fluorescence (DAPI staining) indicates cell nuclei. Quantitative analysis of the red-to-green fluorescence ratio after 24 h (**C**) and 48 h (**D**) pretreatment followed by UVA exposure. Data are expressed as mean ± SD (n = 3). * *p* < 0.05, ** *p* < 0.01, *** *p* < 0.001, **** *p* < 0.0001 versus UVA group; ^##^ *p* < 0.01, ^###^ *p* < 0.001 versus the RFF or SA treatment groups.

Consistent with the restoration of ATP and NAD^+^ levels, mitochondrial function was further assessed by measuring the mitochondrial membrane potential (ΔΨm), a key indicator of mitochondrial integrity [38,39]. Using the JC-1 probe, ΔΨm was evaluated based on the red-to-green fluorescence ratio, with higher ratios reflecting better mitochondrial function. As shown in Figure 5A,B, UVA irradiation markedly decreased this ratio, indicating loss of ΔΨm and impaired mitochondrial function. In contrast, pretreatment with RFF, SA, or their combination for 24 h or 48 h attenuated this decrease, resulting in enhanced red fluorescence and a restored red-to-green ratio, suggesting improved ΔΨm stability. Quantitative analysis (Figure 5C) revealed that after 24 h of pretreatment, the red-to-green ratio increased by 687.7% in the RFF group and 300.0% in the SA group relative to the UVA-exposed group. Combined treatment further elevated the ratio to 1286.5%, which was 87.0% and 328.8% higher than RFF or SA alone, demonstrating pronounced combined effect. After 48 h of pretreatment, the combination treatment further improved ΔΨm protection (Figure 5D), yielding a red-to-green ratio significantly higher than that of the UVA group and 5.3% above the untreated control (*p* < 0.05). These results demonstrate that the RFF–SA combination effectively preserves ΔΨm stability and supports mitochondrial function.

### 3.6. Transcriptomic Analysis Reveals Activation of SIRT and Energy Metabolism Pathways

**Figure 6:**
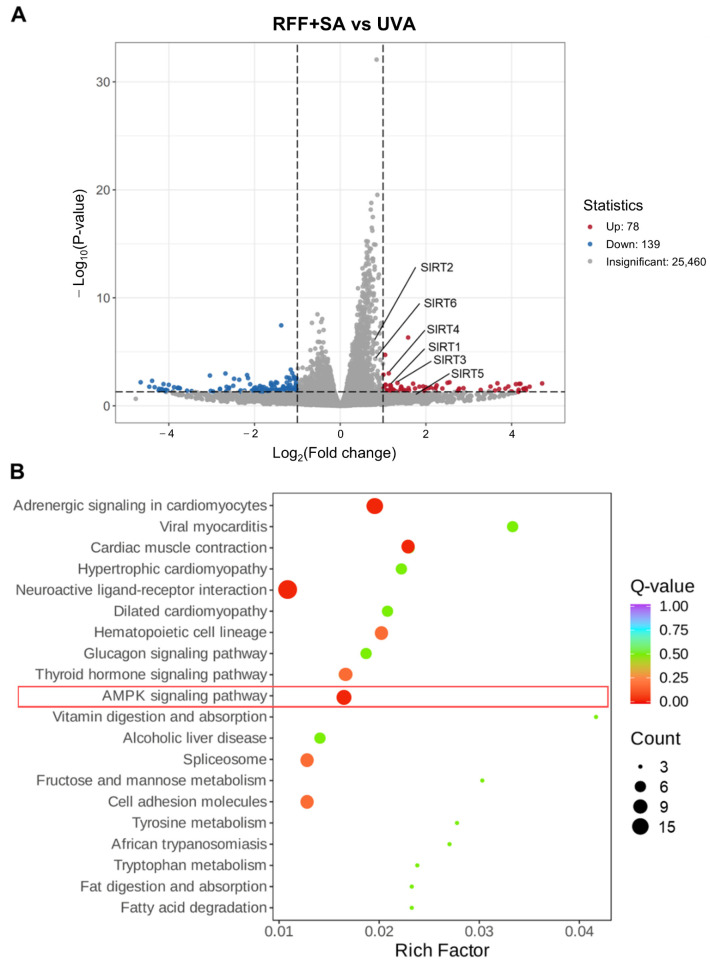
Transcriptomic responses of UVA-irradiated HDF cells to combined treatment with SA and RFF. (**A**) Log_2_ fold changes in the expression of sirtuin family genes in cells treated with RFF+SA compared with UVA-irradiated cells. Dashed lines indicate ±1 log_2_ fold-change thresholds. (**B**) KEGG pathway enrichment analysis of differentially expressed genes (DEGs) identified between the UVA group and the RFF+SA treatment group. Bubble size represents the number of genes enriched in each pathway, and the color gradient indicates the Q-value. The rich factor is defined as the ratio of DEGs in a given pathway to the total number of genes annotated in that pathway.

To further elucidate the molecular mechanisms underlying the observed improvements in mitochondrial function and energy metabolism, RNA-seq analysis was performed on UVA-exposed fibroblasts with or without RFF–SA pretreatment. Differential expression analysis identified 217 significantly dysregulated genes in the combination group compared with the UVA group, including 78 upregulated and 139 downregulated genes (Figure 6A). Among sirtuin family members, SIRT1, SIRT3, and SIRT4 exhibited >2-fold induction (log_2_FC ≈ 1.0–1.3), whereas SIRT5 showed a positive trend that did not reach statistical significance, likely due to low read counts and partial RNA degradation in irradiated samples. These results suggest a pivotal role for NAD^+^-dependent deacetylase activity in the observed protection.

KEGG pathway enrichment analysis revealed that the most significantly modulated pathways were primarily associated with cellular energy metabolism and stress adaptation (Figure 6B). These included the AMPK signaling pathway, a central regulator of mitochondrial biogenesis and energy homeostasis, as well as several cardiomyopathy-related pathways (e.g., cardiac muscle contraction, hypertrophic cardiomyopathy), which involve key genes in calcium handling, contractile function, and bioenergetics. Additional enriched pathways included neuroactive ligand-receptor interaction and glucagon signaling, indicating potential effects on cellular communication and metabolic regulation. Notably, the activation of energy metabolism-related pathways aligns with our experimental observations of enhanced mitochondrial function, supporting the notion that the RFF–SA combination improves mitochondrial bioenergetics and cellular stress resilience.

### 3.7. Validation of SIRT Gene Expression by qPCR

**Figure 7:**
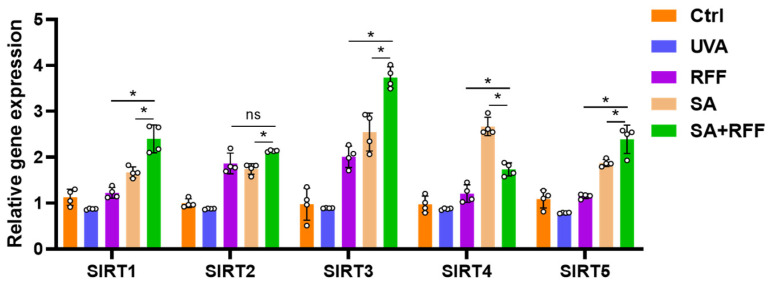
qPCR validation of sirtuin gene expression in HDF cells. Cells were pretreated with RFF, SA, or their combination (RFF+SA) for 24 h or 48 h prior to UVA irradiation. Bars represent the mean ± SD (n = 3). * *p* < 0.05 indicates a significant difference; ns (not significant) denotes no significant difference.

To validate the transcriptomic findings, mRNA levels of key sirtuin family members were quantified by qPCR. Figure 7 demonstrates that UVA irradiation significantly suppressed the expression of SIRT1, SIRT3, SIRT4, and SIRT5. Pretreatment with RFF or SA alone partially restored their expression, whereas the RFF–SA combination produced the most pronounced upregulation across all five tested SIRT genes (SIRT1–5). Notably, the combination treatment not only fully reversed the UVA-induced downregulation but also further increased SIRT1, SIRT3 and SIRT5 expression beyond the levels observed with either single agent. These results are consistent with the RNA-seq data and confirm that the RFF–SA combination enhances SIRT gene expression at the transcriptional level more effectively than either treatment alone.

## 4. Discussion

Skin photoaging, primarily driven by UVA radiation, is characterized by collagen degradation, loss of elasticity, and other structural alterations [40]. UVA penetrates the dermis, where it induces oxidative stress and mitochondrial dysfunction in fibroblasts, leading to impaired bioenergetics, disrupted collagen synthesis, and cellular senescence [41]. Consequently, strategies that simultaneously enhance mitochondrial resilience and reinforce antioxidant defenses are essential for effective photoaging intervention.

**Figure 8:**
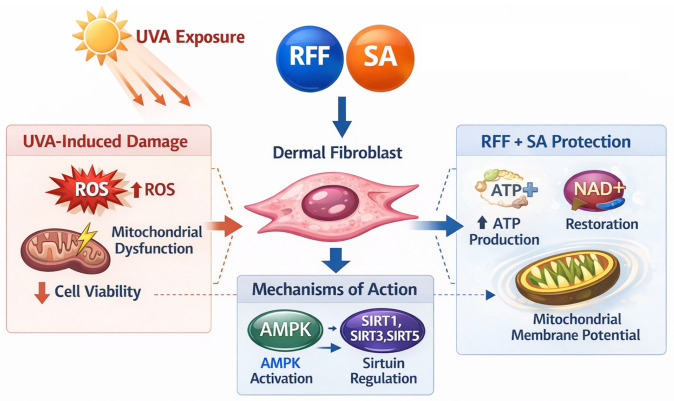
Schematic illustration depicting the protective effects of RFF and SA on dermal fibroblasts against UVA-induced oxidative stress and mitochondrial dysfunction.

The present study demonstrates that the combination of RFF and SA confers robust protection against UVA-induced photodamage in HDF (Figure 8), with greater efficacy than either component alone. Dermal fibroblasts, as primary targets of UVA-induced oxidative stress and mitochondrial dysfunction, provide a relevant model for photoaging. The 24 h and 48 h pretreatment time points were selected to capture both short-term and longer-term cellular responses to UVA stress. A 24 h pretreatment assesses immediate protective effects, particularly on ROS generation and partial stabilization of mitochondrial membrane potential (ΔΨm), whereas 48 h captures cumulative responses, including recovery of mitochondrial energy metabolism (ATP and NAD^+^) and stabilization of mitochondrial dynamics. Specifically, 24 h primarily mitigated early oxidative stress, reducing ROS and partially stabilizing ΔΨm, whereas 48 h reflected cumulative effects through coordinated activation of endogenous repair and energy restoration pathways. The RFF–SA combination enhanced cell viability, attenuated ROS accumulation, preserved ΔΨm, and elevated ATP and NAD^+^ levels, with stronger effects observed at 48 h. It should be noted that ROS was measured using DCFH-DA, which may also detect other oxidative processes; thus, the observed reductions reflect relative decreases in oxidative stress rather than absolute ROS levels [42].

The UVA-induced depletion of NAD^+^ and ATP may involve PARP1, a DNA damage-responsive enzyme that consumes NAD^+^ during repair; its hyperactivation can limit mitochondrial oxidative phosphorylation, exacerbating energy stress [43,44]. While PARP1 activity was not directly assessed, RFF–SA treatment upregulated SIRT1, SIRT3, and SIRT5, NAD^+^-dependent deacetylases essential for mitochondrial function, energy metabolism, and oxidative stress defense, as confirmed by qPCR. By restoring NAD^+^ levels, the combination may counteract PARP1-mediated depletion and enhance SIRT activity, preserving mitochondrial integrity and sustaining ATP production under UVA stress. Transcriptomic analysis further revealed coordinated upregulation of SIRT1–5 and activation of the AMPK pathway, central regulators of metabolism and stress adaptation [45,46]. These results suggest a mechanistic link between NAD^+^ availability, SIRT activation, AMPK engagement, and protection of mitochondrial function, although further experimental validation is warranted.

The superior efficacy of the RFF–SA combination likely arises from complementary multi-target modulation. Rather than acting through a single pathway, RFF and SA influence interconnected processes, whereby their combined antioxidant effects more effectively reduce ROS, while modulation of NAD^+^ metabolism and SIRT activation supports mitochondrial function and energy homeostasis. This coordinated regulation enhances cellular resilience under UVA-induced stress. Both ingredients possess intrinsic antioxidant activity, but their combination produces a pronounced effect, likely via multi-target radical scavenging and amplification of endogenous defenses. More importantly, the combination restores cellular energy status, as reflected by NAD^+^ and ATP preservation, indicating improved mitochondrial function and reduced metabolic stress [47,48]. Transcriptional activation of SIRT genes links these phenotypic improvements to enriched energy metabolism pathways, which were identified by KEGG analysis. Through these mechanisms, RFF–SA may also attenuate UVA-induced fibroblast senescence by supporting mitochondrial function, enhancing DNA repair, and maintaining cellular homeostasis. Collectively, these effects provide a mechanistic basis for the observed cellular protection, although the precise pathways underlying NAD^+^ and ATP preservation require further investigation.

Furthermore, transcriptomic enrichment highlighted the AMPK signaling pathway as a key target of the combined treatment. AMPK is a master energy sensor that activates catabolic processes to generate ATP while inhibiting anabolic pathways under energetic stress [49]. Its activation often cooperates with the SIRT pathway, forming a feed-forward loop that sustains metabolic homeostasis [50]. The observed SIRT upregulation alongside AMPK pathway enrichment indicates a potential coordinated transcriptional response. However, due to the lack of direct functional validation, this association should be interpreted with caution. Nevertheless, these changes may contribute to enhanced mitochondrial bioenergetics and stress resilience, though the precise contribution of AMPK signaling requires further investigation.

While this study provides compelling evidence for the combined photoprotective effects of the RFF-SA combination, several limitations should be considered. First, all experiments were conducted in vitro using a monolayer fibroblast culture, which may not fully replicate the complex cellular interactions, matrix environment, and systemic factors present in intact human skin. Second, the mechanistic insights, though strongly supported by transcriptomic and biochemical data, rely primarily on correlative evidence; direct validation of key pathways (e.g., AMPK signaling) was not performed in this study, and future studies employing genetic or pharmacological inhibition of key targets (e.g., SIRT1/3, AMPK) are warranted to establish causal relationships. Third, the concentrations and treatment durations used here were optimized for the cellular model and may require further adjustment for translation to topical formulations and in vivo efficacy. Finally, long-term safety, bioavailability, and stability of the combination in cosmetic or dermatological products remain to be evaluated in preclinical and clinical settings. Addressing these aspects in subsequent work will strengthen the translational relevance of the proposed mechanism and support the development of RFF-SA-based photoprotective interventions.

In conclusion, this study demonstrates that the photoprotective effects of RFF and SA are mediated by their coordinated action on the NAD^+^–SIRT–AMPK regulatory axis. By simultaneously elevating NAD^+^ bioavailability, activating sirtuin expression, and engaging AMPK-mediated energy-sensing pathways, the combination treatment comprehensively mitigates oxidative stress, reverses mitochondrial dysfunction, and restores cellular bioenergetics. These findings provide a mechanistic rationale for the observed phenotypic superiority of the RFF-SA combination and suggest its potential as a strategic ingredient system in advanced photoprotective and anti-aging formulations designed to preserve dermal fibroblast function and overall skin integrity.

## Data Availability

All the data used to support the findings of this study are available from the corresponding authors upon reasonable request.
